# Ginger: A Nutraceutical Supplement for Protection Against Various Cardiovascular Diseases in Clinical Trials

**DOI:** 10.7759/cureus.80841

**Published:** 2025-03-19

**Authors:** Taherah Mohammadabadi, Aimen E Ben Ayad, Akhil Maheshwari

**Affiliations:** 1 Faculty of Animal Science and Food Technology, Agricultural Sciences and Natural Resources University, Mollasani, IRN; 2 Department of Newborn Health or Neonatology, Global Newborn Society, Newborn, Clarksville, USA; 3 Department of Pediatrics/Neonatology, Tawam Hospital, Al Ain, ARE; 4 Department of Pediatrics, United Arab Emirates University, Al Ain, ARE; 5 Department of Neonatology/Pediatrics, Boston Children's Health Physicians/New York Medical College, New York, USA; 6 Department of Pediatrics/Neonatology, Banaras Hindu University Institute of Eminence, Varanasi, IND

**Keywords:** atherosclerosis, ginger, gingerols, hyperlipidemia, hypertension, nutraceutical, phytochemical

## Abstract

Cardiovascular diseases (CVDs) are increasing in prevalence, causing significant health issues and remaining one of the leading causes of death worldwide. Medical herbs continue to be used as an alternative treatment approach for several diseases, including various CVDs. Since ancient times, certain herbs have been safely used to alleviate the risk of developing CVD and control or improve the symptoms of medical conditions, such as in cases of congestive heart failure, angina, atherosclerosis, and systolic hypertension. Ginger is one of the medicinal herbs that neutral agents use to prevent and treat various CVDs. Ginger has antioxidant, anti-inflammatory, and immunomodulatory components and may improve cardiovascular risk factors. The natural components of ginger effectively inhibit inflammation, oxidative stress, and insulin resistance; may reduce fasting blood glucose, triglyceride, and low-density lipoprotein (LDL) levels; and prevent CVDs. Ginger can be an alternative that has lower side effects. Ginger's bioactive components may improve human blood lipid profile and decrease blood sugar levels. Further research is necessary to confirm ginger phytochemicals' efficacy and mechanism for various CVDs. The present review aims to summarize the effects of ginger's bioactive compounds on cardiovascular diseases.

## Introduction and background

The global impact of death from cardiovascular diseases (CVDs) is staggering, with approximately 80% attributed to heart attacks. By 2030, this number is expected to exceed 22.2 million people [1]. Notably, 7.4 million deaths were due to coronary heart issues, and 6.7 million were due to stroke [2]. Women, in particular, aged between 25 and 59 years, face a higher mortality rate from cardiovascular problems, coronary and aortic diseases, and heart failure [3,4]. The conditions of the heart and blood vessels lead to CVDs such as heart attacks, stroke, congenital heart failure, peripheral artery and rheumatic heart issues, and hypertension [2]. Common risk factors for CVDs include diabetes, hypertension, unhealthy diet, hyperlipidemia, inflammation, and smoking [5]. It has been established that diabetes or hypertension increases inflammation and causes CVDs [6]. Phytochemicals play a significant role in preventing CVDs. Compared to saturated fats, high poly- and monounsaturated fats have been shown to be cardioprotective [7]. Increased phytochemical and plant polyphenol intake may lower the low-density lipoprotein (LDL)/high-density lipoprotein (HDL) ratio, a marker of CVD [8]. Phytochemicals are naturally occurring chemical compounds that plants produce. The term "phytochemical" comes from the Greek word "phyto," which means "plant." Phytochemicals are used for atherosclerosis, congestive heart failure, arrhythmia, and systolic hypertension [2]. However, prolonged use or overdose of some herbs, for example, licorice, *Digitalis*, yohimbe, and kava, may lead to some side effects, particularly an increased risk of cardiovascular issues like high blood pressure and tachycardia [9]. The natural components of phytochemicals, including carotenoids, polyphenols, tocotrienols, catechin, quercetin, resveratrol, sulforaphane, isoflavones, and flavonoids, effectively inhibit inflammation and oxidative stress, thereby preventing CVDs [10]. Ginger, the rhizome of *Zingiber officinale Rosc.*, is a rich source of bioactive compounds that relate to the Zingiberaceae family and are grown mainly in India, China, and Southeast Asia. Ginger is extensively cultivated in the southeast region of Asia. The Arabs transported it from India to East Africa, while the Portuguese spread it to West Africa. Tropical and subtropical climates are proper for ginger cultivation, and it cannot tolerate very low temperatures. Ginger comprises carbohydrates (60%-70%), proteins (9%), lipids (3%-6%), ash (8%), crude fiber (3%-8%), and essential oil (2%-3%) [11]. More than 150 wild and cultivated zingiberaceous species have been reported. The main commercially cultivated species are *Z. officinale*, *Curcuma longa*, and *Alpinia galanga* [12]. The bioactive compounds in ginger, including gingerols, paradol, and shogaol, contribute to its antioxidant and anti-inflammatory properties [13,14]. More than 400 bioactive compounds have been identified in ginger, with the therapeutic value primarily dependent on gingerols, shogaols, curcumin, paradols, and terpenoids, and 6-gingerol is the most abundant component [15]. Conventional methods for ginger extraction include heat reflux, distillation, percolation, and Soxhlet extraction. Advanced methods include microwave-assisted extraction, pressure liquid extraction, and ultrasonication or ultrasound-assisted extraction [16]. Ginger extract inhibits lipid peroxidation due to its antioxidant effects, preventing atherosclerosis, improving lipoprotein, reducing lipogenesis, and preventing cardiovascular complications. It has been confirmed that consuming 2 g of ginger for 12 weeks reduced inflammation, insulin resistance, fasting blood glucose, and triglyceride (TG) without impacting total cholesterol (TC), HDL, or LDL levels [17]. This review, supported by scientific literature, explores ginger's efficacy in CVD.

## Review

Cardioprotective property of phytochemicals

Phytochemicals like polyphenols and flavonoids are cardioprotective against various cardiovascular issues due to their antioxidant and anti-inflammatory properties [18]. They can reduce serum lipids, interact with calcium channels, and inhibit platelet formation. Ginger's phenolic compounds may reduce high blood pressure by a similar mechanism to calcium channel blockers, common antihypertensive medications [19]. Phytochemicals can also improve atherosclerosis, lipid deposition, and oxidative stress [4,20]. Polyphenols, flavonoids, and other phytochemicals have shown promising potential in managing hyperlipidemia by lowering TC, reducing LDL and TG levels, potentially inhibiting cholesterol biosynthesis, and increasing bile acid excretion in addition to regulating apoptosis in the endothelium [21]. Flavonoids' antioxidant and anti-inflammatory functions may offer protection against atherosclerosis, suppressing the production of pro-inflammatory cytokines and reducing platelet aggregation, key processes in plaque formation. In addition, it reduces mortality and stroke, encouraging further research and application [4]. Quinones, such as thymoquinone, found in some herbs, such as black seed (*Nigella sativa*), are phytochemicals with cardioprotective properties. They enhance mitochondrial function, the process by which cells generate adenosine triphosphate (ATP), thereby increasing the heart's energy supply [22]. Plant sterol may reduce TC and LDL by 15% and improve CVD [23]. Some herbs contain alkaloids, including certain acridones, indoles, imidazoles, and purines. These alkaloids and other phytochemicals like polyphenols and terpenes may contribute to cardiovascular health by neutralizing harmful free radicals, reducing oxidative stress, and dampening inflammation-key factors in developing atherosclerosis and other heart conditions [4]. Plant sulfur is a phytochemical that can activate Nrf2, a master regulator of the body's antioxidant and detoxification pathways. It can also inhibit cholesterol synthesis. Sulforaphane has antioxidant and anti-inflammatory properties. Allicin has antiplatelet effects and inhibits cholesterol synthesis, which prevents CVD atherosclerosis [24]. These compounds reduce the severity of atherosclerosis, inhibit platelet aggregation, and reduce blood pressure [25].

Bioactive compounds of ginger

**Figure 1:**
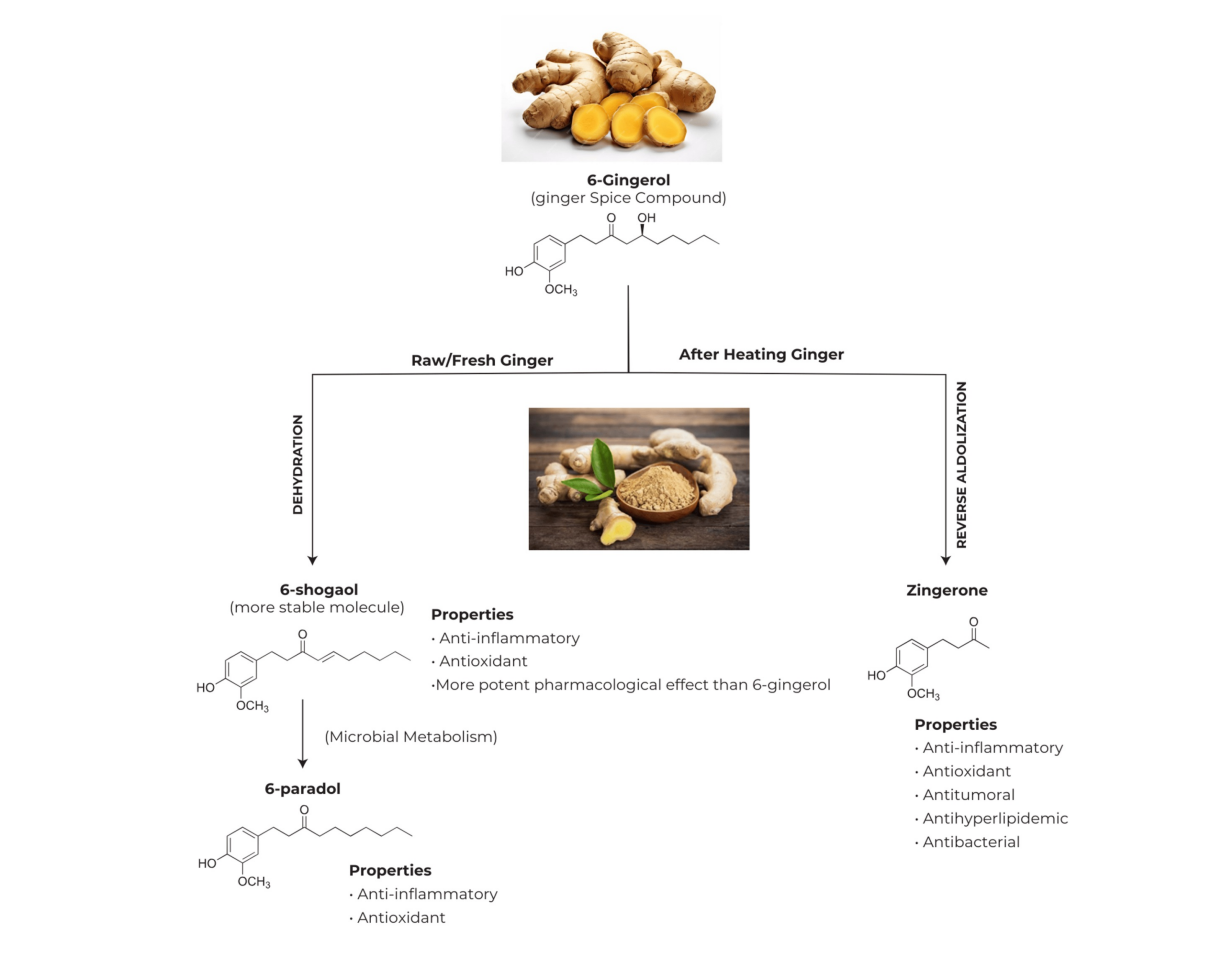
Metabolization of 6-gingerol Mechanism of effects of 6-gingerol on cardiovascular disease Created by Aimen E. Ben Ayad

It is proven that ginger (*Z. officinale*) has cardioprotective effects due to its antioxidant, antiplatelet, and antihypertensive properties [26,27], which are mostly due to terpenes and phenolic compounds [28]. Ginger terpenes such as α-zingiberene, camphene, ar-curcumene, β-phellandrene, β-bisabolene, and α-piene have antioxidant, anti-inflammatory, and antidiabetic properties [29]. Four phenolic components, gingerols, shogaols, parasols, and zingerone (ZGR), among 400 various components in ginger, are mostly responsible for its therapeutic properties [29]. 6-Gingerol is the most abundant component in fresh ginger, which can change into 6-shogaol by long storage and dehydration [30,31]. In vitro studies found that 6-shogaol is more non-reactive and has higher therapeutic characteristics than 6-gingerol. 6-Shogaol could convert to 6-paradol with the same antioxidant and anti-inflammatory effects [30,32]. ZGR is synthesized by reverse aldolization of gingerols following heating fresh ginger, which has anti-inflammatory, antioxidant, and antiepidemic activity [30,33,34]. The highest 6-gingerol amount was in the methanol extract of *Z. officinale* root (17.09 mg g^−1^ extract) and ginger powder (15.92 mg g^−1^ extract) [15,30]. Oral (70-140 mg/kg) or intravenous (1.75-3.5 mg/kg) consumption of 6-gingerol and 6-shogoal decreased BP in the cell-based assay or cell culture. 6-Gingerol may be considered a novel angiotensin II type 1 receptor antagonist. The aqueous extract of ginger (0.05 mg/mL) prevents lipid peroxidation and angiotensin-converting enzyme (ACE) in rat hearts; reduces TC, LDL, TG, very-LDL (VLDL), and ZGR; and can scavenge oxidants and free radicals [15]. The metabolization of 6-gingerol is given in Figure 1.

Ginger dosage

The various results obtained from different studies are linked to the variety, purity, and bioactive components of ginger, as well as the processing method and the amount of ginger. Ginger was usually considered safe in the FDA-approved list at dosages of up to 4 g/day [35]. The daily consumption of 3 g of ginger powder/day for 6-12 weeks in type 2 diabetes patients has no adverse effects [36]. Higher doses of ginger, more than 6 g, can cause a few minor adverse effects, such as heartburn, abdominal discomfort, and mild diarrhea [35]. There is an association between ginger and spontaneous abortions in pregnant women. It is confirmed that consumption of ginger in pregnant women reduced nausea and vomiting, headache, abdominal discomfort, arrhythmia, intolerance, diarrhea, allergic reaction, heartburn, dizziness, and drowsiness [37]. Ginger administration of up to 4 g/day has not affected platelet aggregation [35]. Ginger powder was administrated in 500, 1,000, and 1,500 mg/day for three and two months and six weeks, respectively, in human studies. Also, immunomodulatory doses were 1 μg/mL to 10 mg/mL for ginger extract, 6.5-100 μM for 6-gingerol, and 25-100 μM for 8- and 10-gingerol. The effective immunomodulatory doses ranged from 28 to 720 mg/kg/day for ginger extract and 10 and 50 mg/kg/day for 6-shogaol in mice. The effective anti-inflammatory and antioxidative doses ranged from 200 to 500 mg/kg/day of ginger extract [15].

Overview of the beneficial impacts of ginger

Extensive research has found that ginger possesses a wide spectrum of anti-inflammatory, immunomodulatory, and antioxidative properties. These properties have been observed in various health conditions, such as osteoarthritis, rheumatoid arthritis, type 2 diabetes, respiratory distress, liver disease, and neurological disorders like epilepsy, Alzheimer's, migraine, and Parkinson's. The anti-inflammatory effects of ginger or its components were reported on airway inflammation, lipopolysaccharide (LPS)-mediated liver failure [38], ulcerative colitis, neuroinflammation, neuropathic pain, fibromyalgia syndrome [39], and peptic ulcers in animal models. Ginger, a herb with a long history in traditional medicine, has been used for centuries to treat various ailments. Its applications range from relieving arthritis, stomachache, diarrhea, and nausea to addressing respiratory disorders and toothache. The herb's effectiveness in improving immunological and inflammatory issues has been well documented in traditional medicine. According to some studies, no significant adverse effects (except platelet aggregation and probably abortions) have been concluded for ginger. However, the impact of ginger on platelet aggregation requires further clinical trials for confirmation [37]. Additionally, more studies are required to investigate the side effects of high doses or long use of ginger or its bioactive components [15]. Ginger extract consumption inhibits lipid peroxidation due to its antioxidant effects, preventing atherosclerosis and improving lipoprotein [27]. It prohibits chemotherapy-induced emesis, nausea, and respiratory diseases [40]. 6-Gingerol regulates lipogenesis, fatty acid oxidation, oxidative stress, and melanogenesis in murine melanoma cells of aging rats due to antioxidant properties. Ginger increases antioxidant enzyme functions, such as superoxide dismutase, and decreases malondialdehyde (MDA), a lipid peroxidation marker, in rats in a dose-dependent manner [41]. Ginger heightens lipolysis, reduces lipogenesis, and controls obesity, a risk for cardiovascular complications [27].

Effects of ginger on CVDs

Anti-inflammatory Effects

Arterial inflammation is a risk factor for heart disease due to oxidized arachidonic acid (AA) compounds like lipoxygenase and cyclo-oxygenase. It has been proved that ginger components prevent the production of inflammatory cytokines, nitric oxide (NO), prostaglandin synthase, and arachidonate-5-lipoxygenase in a dose-dependent manner [42]. In chronic inflammation, pro-inflammatory factors, like cytokines and prostaglandins, are released and cause the development of autoimmune diseases, CVDs, and cancer [43,44]. Ginger significantly lowers TNF-α, inflammatory factors, and NO synthase. It also reduces different pro-inflammatory markers and inhibits pro-inflammatory cytokines. The most active anti-inflammatory compounds are 6-shogaol, 6-gingerol, and 6-dehydroshogaol of ginger [45,46]. Ginger presents a novel approach to managing CVDs due to its inhibitory action against inflammation and oxidative stress. It also inhibits COX-1 and COX-2, reduces prostaglandin synthesis, and minimizes the production of pro-inflammatory factors like PGE2, IFN-γ, TNF-α, and IL-6 [14,47].

Antiobesity Activity

Obesity has deleterious effects, mainly on CVDs, fatty liver disease, hypertension, hyperlipidemia, and cancer [27]. Gingerenone-A has a higher inhibitory effect on lipid accumulation and adipogenesis than gingerols and 6-gingerol, which appears to activate fatty acid metabolism [48]. The daily 2 g of ginger powder in obese women decreases body mass index and increases fat oxidation in humans [49]. Therefore, ginger and its bioactive components are against obesity by increasing lipolysis and inhibiting adipogenesis [27]. Studies on the effects of 6-gingerol on obesity have been conducted on both animals and humans. One study used 6-gingerol (25, 50, and 75 mg kg^−1^) daily for 30 days in obese mice. The results showed a significant reduction in body weight as well as a decrease in tissue lipids, glucose, leptin, and insulin levels [50]. Maharlouei et al. found that weight and fasting glucose decreased and HDL cholesterol increased using ginger without affecting insulin, TGs, TC, or LDL [51]. Ginger's ability to increase thermogenesis and lipolysis, suppress lipogenesis, control intestinal absorption, and moderate obesity makes it a promising therapy for obesity and its related complications [52].

Antioxidant Activity

Ginger has great antioxidant activity, increases antioxidant enzymes, decreases oxidative stress, and eradicates free radicals [52] in cancer patients [27,53,54]. 6-Gingerol, 8-gingerol, 10-gingerol, and 6-shogaol in ginger exhibit antioxidant activity, and the highest antioxidant activity is for 6-gingerol, followed by 6-shogaol [55]. 6-Gingerol inhibits xanthine oxidase and the production of reactive oxygen species, raising the activity of antioxidant enzymes superoxide dismutase and catalase [53]. The harvest time affects ginger's antioxidant function; the antioxidant activity is higher in early harvest, from week one to the first month [56]. The antioxidant effect of ginger is almost equal to that of ascorbic acid [57]. Ginger's ability to scavenge free radicals lowered lipid peroxidation and protected cell membranes from oxidation, increasing the levels of antioxidant enzymes and glutathione in a dose-dependent manner [42].

Antidiabetic Activity

Diabetes is a metabolic disorder with increased blood glucose [58]. The administration of 6-gingerol modulates glycogen synthase 1 and glucose transporter type 4 (GLUT-4) to the cell membrane, increasing glucose entry in skeletal muscles [59]. It proved that 1,600 mg of ginger daily versus wheat flour for 12 weeks reduces fasting plasma glucose, insulin, glycosylated hemoglobin (HbA1c), TC, and TGs in 70 type 2 diabetes patients [27]. 6-Gingerol inhibited fasting blood glucose and enhanced glucose intolerance, reducing insulin resistance and lipid profile in type 2 diabetes [15]. Gingerol also showed protective properties against CVDs [60].

Effect of Ginger on Lipid Profile

Impaired blood lipid levels contribute to CVD. Ginger's varying effects on blood lipid profiles are related to many factors, such as dosage, ginger form, duration, experimental model, population, ginger variety, environmental conditions, and harvest time. Ginger decreases TGs and LDL without significantly reducing TC. Daily consumption of less than 2 g of ginger powder is more effective in lowering TGs and cholesterol [59]. Since it is safe, it will improve the lipid profile and prevent CVD. Doses of up to 1.8 g/day significantly reduce TG, TC, LDL cholesterol, and LDL/HDL ratio in obese patients treated with metformin [60]. Ginger did not show any effect on blood lipids and body composition without any significant differences in cholesterol lipoproteins [61], TGs, cholesterol, or HDL [27]. Consuming 2 g of ginger daily for 12 weeks reduced apolipoprotein B and the apolipoprotein B/apolipoprotein A-I ratio, increasing the apolipoprotein A1 ratios [27]. Ginger significantly increases HDL and lowers TGs and cholesterol (hyperlipidemia and type 2 diabetes mellitus (T2DM)). Less than 2 g of ginger consumed daily for 50 days reduces TC and TG [59]. Ginger components inhibit intestinal lipase enzymes, fat hydrolysis, and cellular cholesterol synthesis and increase hepatic cholesterol 7α-hydroxylase that converts cholesterol into bile acids [3].

Antiplatelet Aggregation Activity of Ginger

The different effects of ginger on platelet function are related to various factors as well, such as differences in dosage use, ginger form, duration, experimental model, population, ginger variety, environmental condition, and harvest time. ZGR in ginger has antithrombotic and anticoagulant effects. ZGR inhibits platelet aggregation and reduces activated thromboplastin time [27]. Consumption of 10 g powdered ginger after four hours significantly reduced adenosine diphosphate (ADP)- and adrenaline-induced platelet aggregation in humans. 6-Gingerol and 6-shogaol showed the most function against cholesterol, AA, thrombin, and platelet aggregation [62]. 6-Paradol, 10-dehydrogingerol, and 10-gingerol had the most significant inhibition on AA-induced aggregation. Ginger aqueous extract decreased TG, thromboxane-B2 cholesterol, and PGE2, increasing adenosine levels in the rats. So, it improves vasodilatation, reduces hypertension, and prevents platelet aggregation. Five grams of ginger a day significantly reduced platelet aggregation in healthy humans with high-fat foods. However, a daily dose of 4 g for three months was ineffective in treating coronary artery disease [42].

Ginger Against Blood Pressure

Ginger's effects on blood pressure can also vary based on dosage, ginger form, duration, experimental model, population, variety of ginger, environmental conditions, and harvest time. Inflammation is linked with cardiovascular issues, which results in hypertension, a strong risk factor for CVD [27]. 6-Shogaol and 9-gingerol in ginger are responsible for antihypertensive effects, which reduce cholesterol and LDL, reduce atheroma plaque formation, and increase vessel elasticity [63]. The antihypertensive effect of doses ≥ 3 g/day of ginger in ≤50 years, during ≤8 weeks, reduced the probability of ischemic heart disease and hypertension [42,64]. Ginger has a dose-dependent hypotensive effect, causes vasodilation in rats and rabbits, induces vasoconstriction, and exhibits calcium channel-blocking activity similar to verapamil [65]. The impact of ginger on lipids is shown in Table 1.

**Table 1:** The effect of ginger on the lipid profile in different trials BMI: body mass index; TC: total cholesterol; LDL: low-density lipoprotein; TG: triglyceride; HDL: high-density lipoprotein; T2DM: type 2 diabetes mellitus; ApoB: Apo protein B; Apo A1: Apo protein A1

Cases	Ginger dosages	Results	Reference
Obese Egyptian patients with T2DM (n = 80), 30–60 years	600 mg, 3 times/day, 8 weeks	↓ BMI, ↓ TC, ↓ LDL, ↓ TG, ↑ HDL	Alizadeh-Navaei et al., 2008 [66]
Non-diabetic hyperlipidemia patients (n = 45 ginger, 40 placebo)	3 g/day, 45 days	↓ TC, ↓ TG	Arablou and Aryaeian, 2018 [67]
Obese men (n = 32), 18–30 years	1 g/day, 10 weeks	ns. TC, TG, LDL-C, HDL-C, ↓ TC	Atashak et al., 2011 [68]
T2DM patients (n = 64), 38–65 years	2 g/day, 8 weeks	↓ LDL, ↓ TG	Makhdoomi Azrati et al., 2017 [61]
T2DM patients (n = 63), 20–60 years	1.6 g/day, 12 weeks	↓ TC, ↓ TG	El Gayar et al., 2019 [60]
T2DM (n = 50), 20–60 years	2 g/day, 12 weeks	↓ ApoB, ↓ ApoB/Apo A1 ratio, ↑ Apo A1	Khandouzi et al., 2015 [69]
Hyperlipidemic patients (n = 100), 35–60 years	3 g/day, 30 days	↓ TC	Mazidi et al., 2016 [70]
T2DM (n = 50)	2 g/day, 10 weeks	↓ LDL/HDL ratio	Pourmasoumi et al., 2018 [59]

## Conclusions

Ginger contains numerous bioactive compounds that decrease inflammation and free radicals and inhibit platelet aggregation. Ginger administration can help lower blood pressure, improve glycemic control, enhance vascular health, and prevent obesity. In addition, it could also improve lipid profile by decreasing TC, LDL, and TG and increasing HDL. Due to ginger's benefits and fewer adverse effects, studies have shown ginger may offer a safer therapeutic approach when used with cardiovascular medications. Therefore, clinical trials suggest that, due to the biological functions and cardioprotective properties of ginger and its constituents, it may serve as a new therapeutic agent for various CVDs.
